# The role of parental health and distress in assessing children’s health status

**DOI:** 10.1007/s11136-022-03186-z

**Published:** 2022-07-25

**Authors:** Sherrie H. Kaplan, Marilou Shaughnessy, Michelle A. Fortier, Marla Vivero-Montemayor, Sergio Gago Masague, Dylan Hayes, Hal Stern, Maozhu Dai, Lauren Heim, Zeev Kain

**Affiliations:** 1 Health Policy Research Institute, University of California, 100 Theory Ste 110, Irvine, CA 92697 USA; 2 Department of Medicine, University of California, Irvine, USA; 3 Newton Wellesley Hospital, Newton, MA USA; 4 Sue & Bill Gross School of Nursing, University of California, Irvine, USA; 5 Department of Psychology, University of Nebraska-Lincoln, Lincoln, NE USA; 6 Donald Bren School of Information and Computer Sciences, University of California, Irvine, USA; 7 Independent Animation Consultant, New York, NY USA; 8 Department of Statistics, University of California, Irvine, USA; 9 Microsoft, Bellevue, WA USA; 10 Department of Anesthesiology & Perioperative Care, University of California, Irvine, USA; 11 UCI Center on Stress & Health, University of California, Irvine, Irvine, USA; 12 Department of Pediatric Psychology, Children’s Health Orange County, Orange, USA; 13 Department of Pediatrics, Children’s Health Orange County, Orange, USA

**Keywords:** Children’s health, Children’s self-reported health-related quality of life, Parent proxy reporting, Parental distress

## Abstract

**Purpose:**

The purpose of the study was to examine the contributions of parents’ health and distress to parent’s and children’s assessments of children’s health.

**Methods:**

We used baseline data from a longitudinal study of 364 children (ages 4–12) about to undergo surgery and their parents in a Southern California pediatric hospital. We used the 20-item child self-reported CHRIS 2.0 general health and the parallel parent-reported measure of the child’s health, along with a measure of parental distress about the child’s health were administered in the perioperative period. Other measures included parents’ physical and mental health, quality of life, distress over their child’s health, and number and extent of other health problems of the child and siblings.

**Results:**

On average, parents’ reports about the child were consistently and statistically significantly higher than children’s self-reports across all sub-dimensions of the CHRIS 2.0 measure. Parents’ personal health was positively associated with their reports of the child’s health. More distressed parents were closer to the child’s self-reports, but reported poorer personal health.

**Conclusion:**

Parent–child differences in this study of young children’s health were related to parental distress. Exploring the nature of the gap between parents and children in assessments of children’s health could improve effective clinical management for the child and enhance family-centered pediatric care. Future studies are needed to assess the generalizability of CHRIS 2.0 to other health settings and conditions and to other racial/ethnic groups.

**Supplementary Information:**

The online version contains supplementary material available at 10.1007/s11136-022-03186-z.

## Plain English summary

Although many studies have found differences between parents’ and children’s views of the child’s health, the reasons for those differences have been less well studied. Using an animated, computer-administered method to ask young children (ages 4–12) questions about their general health (called the Child Health Rating Inventories or “CHRIS”) we compared their answers to those of their parents in a group of children about to undergo surgery (*n* = 364). We found that children gave reliable answers to CHRIS questions, and that parents rated children as healthier than their children did. We also found that parents’ reports about their children were closely related to what they said about their own health. Distressed parents were closer to their children’s self-reports but they also reported poorer personal health. We concluded that the CHRIS measure offers an opportunity to include the child’s voice and a potentially different perspective on children’s health for use by clinicians and researchers. We also concluded that exploring parent–child differences particularly for distressed parents, who may be more ‘vigilant’ observers of children’s health but at a cost to their own health, could provide valuable insight for clinicians caring for these children and their families. Specifically, improved awareness of the perspectives and experiences of both parents and especially children could identify reasons for discrepancies between the two and therefore improve care at home and outcomes after surgery, parent–physician–child communication, and address parents’ worry or distress over their child’s health after surgery.

## Introduction

A number of studies have documented substantial differences between reports of children’s health by parents vs. by children themselves [1–7]. Although the direction of these differences varies somewhat by the disease or condition under study and the child’s age and race/ethnicity, the correspondence between parent and child has been consistently low to moderate [1–7].

Although a number of hypotheses have been advanced for the lack of agreement between parent and child, [1–3, 8] the reasons for those disagreements and the implications for pediatric practice are less well empirically studied [2, 4, 5]. Some studies suggest that parents may be closer to the child’s self-reports for measures involving more observable behaviors, such as those related to physical vs. mental health [4]. Others have suggested that parent–child differences may be narrower for younger children [5, 9]. Still other studies suggest that parents may use their own health as a reference when reporting their child’s health [5].

Some subgroups of parents (e.g., those with children who have chronic conditions) who regularly or closely monitor their children’s health may be closer to the child’s self-perceptions, thus reducing the gap between parents’ and children’s reports [1–7]. Such monitoring or “parental vigilance,” however, may come at a price. Some research suggests that among children with chronic diseases, closer parental monitoring, while improving treatment outcomes, may lead to greater parental distress and poorer personal health [10, 11].

A first step in identifying contributors to parent–child differences in assessments of children’s health is to avoid the potential bias introduced by proxy reporting, particularly for young children [12, 13]. We have developed and previously tested a measure of health-related quality of life specifically designed for independent self-reporting by young children (ages 4–12) [14, 15]. This 20-item measure, the child health rating inventories (CHRIS) [14, 15], uses computer-administered animation to display and record item content and responses, in eight health-related quality of life dimensions scored in real time.

The purpose of the current study was to examine the contributions of parental health and distress to parents’ and children’s self-assessments of the child’s health. In a population of children about to undergo outpatient surgery or minor surgical procedures, we used the CHRIS 2.0 to measure the children’s self-reported health along with parents’ reports of their own health and their distress over the participating child’s health. We also included characteristics previously observed to contribute to parent–child differences, including the child’s age, race/ethnicity, gender, and illnesses, as well as parents’ age, education, race/ethnicity, and gender, family size, and extent of illness among the child’s siblings [2–6, 16–18]. We hypothesized (1) that, based on the literature and our prior work, there would be discrepancies between parents’ and children’s reports in this population and (2) that, adjusted for the characteristics listed above, parents who reported greater distress over the child’s health would report poorer health for the child and for themselves as care givers. We report here the results of that study.

## Methods

### Study design

This is a cross-sectional study reporting baseline data for 364 parent–child dyads participating in a longitudinal cohort study of children ages 4 to 12, undergoing outpatient surgery, described in detail elsewhere [14].

### Site and sample

The study was conducted at a 232-bed pediatric hospital serving a largely poor and minority population in Southern California. Children and their parents were sampled from outpatient surgical logs screened weekly for eligibility from September 1, 2015 through August 30, 2017. Eligibility criteria determined from the child’s medical record and confirmed by parents where relevant were children ages (4–12 years); surgical procedure was either outpatient but not an emergency (defined as urgent surgery not scheduled in advance) or associated with a cancer diagnosis; pre-operative status of the American society of anesthesiologists (ASA) classification of I (a normal healthy patient) or II (a patient with mild systemic disease) (see: https://www.asahq.org/standards-and-guidelines/asa-physical-status-classification-system); no developmental delays or special needs; and both parent and child spoke English or Spanish. Eligible families were offered participation in the study, consented, enrolled, and completed the CHRIS 2.0 child and parent measures 3–7 days prior to surgery. Participating families were emailed or texted electronic links to complete the survey online. Reminders were sent by phone or text to those who had not responded 3 days prior to surgery. The analytic sample included 364 parent–child dyads with complete baseline data for the study variables included in this study.

### Study measures

The CHRIS measures have been described in detail elsewhere [14, 15, 18, 19]. In brief, the updated CHRIS 2.0 measure for children was administered by computer. The 20 items representing eight dimensions of health and response options were “read” to the child by a previously recorded narrator. A frozen frame of each animated five-level Likert-type response option appeared at the bottom of the screen for each item, and the child touched the response that most closely matched their current health state. Each response was recorded and stored in an encrypted database via a secure platform. We have provided sample item content with a static image below.
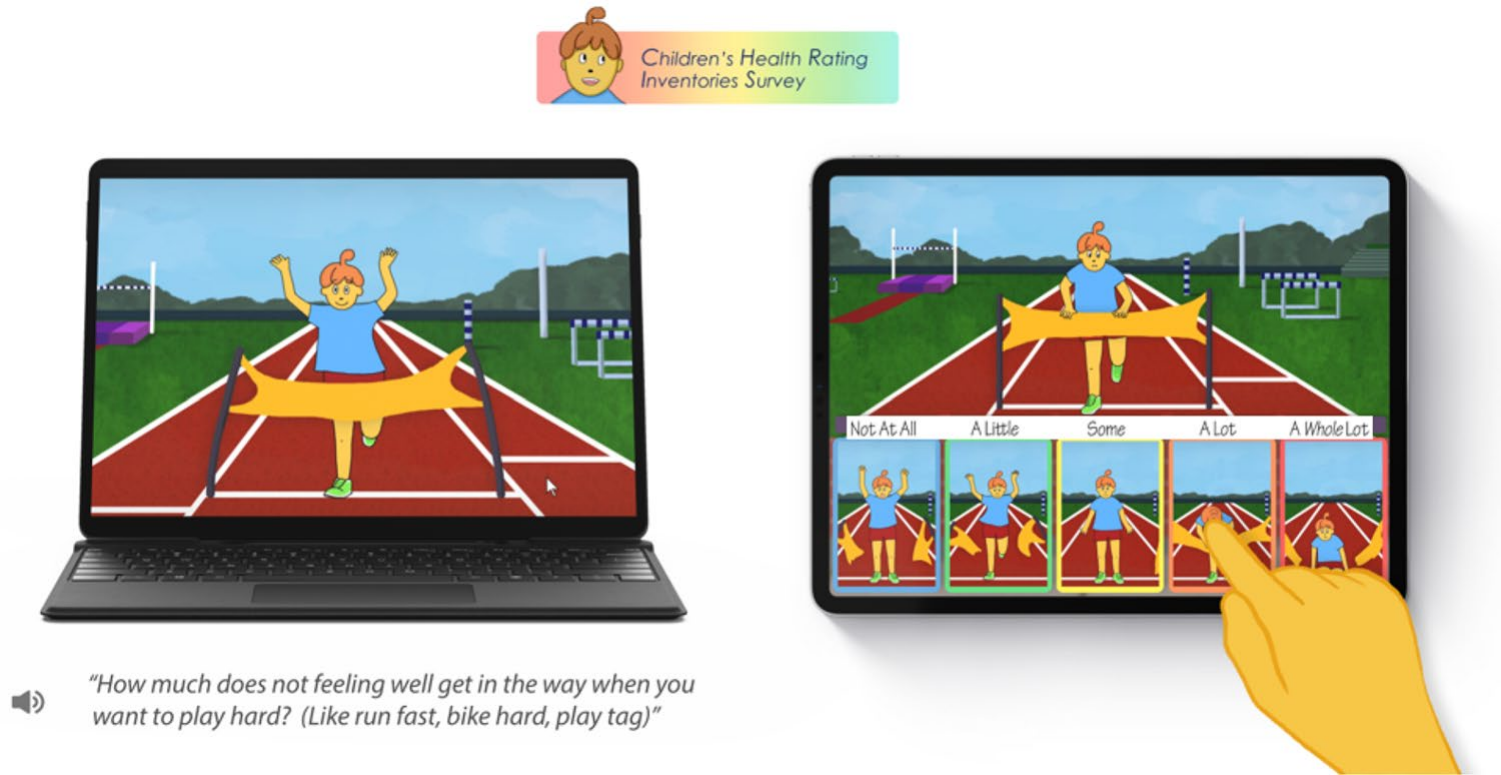


A more complete display of item content, with detailed psychometric analyses appears elsewhere [14]. Based on results from factor analyses [14], a physical health composite was created from 12 items representing physical, role, cognitive, and social function and a mental health composite of 8 items representing mental health, pain, energy, and overall quality of life was created. We also created an overall composite that included all 20 items. CHRIS 2.0 parent questionnaires with parallel content were completed online using the survey platform Qualtrics [20]. All scales have been transformed to range from 0 to 100.

We used 4 items from the CHQ-Parent Form 28 (CHQ-PF28) [21] to assess parental distress, specifically “During the past four weeks, how often did your child’s physical health cause you: emotional suffering or worry?”; limit the amount of time you had for your own “personal needs?” and the same 2 items were repeated for the child’s emotional health. The responses were rated on a five-point Likert scale from “all of the time” to “none of the time.” Parents’ reports about their own physical function, role function, social function, cognitive function, and energy/vitality were measured using items paralleling those from the child-reported CHRIS 2.0, rated on a five-point Likert scale and transformed to range from 0 to 100. Parents’ mental health was assessed using the 5-item Mental Health Index of the Short Form 36 (SF-36) [22]. Overall quality of life, reported by the parent for the child and for their own quality of life, was measured by the 8-item quality of life rating scale (QLRS), with individual items representing each of eight areas of life (work/school life, family life, friendships, daily routine, physical health, mental health, personal/“playtime,” or fun and general life enjoyment), also rated on a 5-point Likert scale from “excellent” to “poor” [23]. Parents were also asked to report the number of the child’s siblings, the number and extent of sibling illnesses, and the participating child’s illnesses (measured as a composite of the presence and severity of 12 chronic conditions—diabetes, thyroid disease, growth problems, obesity, underweight, asthma, seizures, allergies, bowel disease, ADHD, developmental delays, and any other illnesses).

### Statistical approach

Data were analyzed using R [24] and SAS software [25]. We used a variety of summary measures (means, standard deviations, kurtosis, skew) to assess the distributional shape of responses to individual items and scales. We used Cronbach’s alpha [26] to assess internal consistency reliability and factor analysis with varimax rotation to replicate hypothesized scale structure for the CHRIS 2.0 measures (as described elsewhere) [14]. Paired *t* tests were used to assess differences in the mean responses of parents and children on the CHRIS 2.0 measures. One-way random effects intraclass correlation coefficients provided a quantitative measure of the degree of agreement between parent- and child-reported CHRIS 2.0 measures. Parents’ reports about their own health were compared with their reports about their children using Pearson product–moment correlation coefficients. The contribution of parent, child, and family characteristics to differences between parents’ and children’s reports about the child’s health for the physical, mental, and overall composite measures were estimated using separate parsimonious linear regression models for each composite.

Mean differences between parent- and child-reported CHRIS 2.0 measures for most, moderately, and least distressed parents were compared using separate analyses of covariance, adjusting for parental age, education, race, number of siblings in the family, sibling illnesses, child’s age and number of health problems, and types of surgery. Comparisons of the parents’ physical health, mental health and overall quality of life for those parents who were in the highest quartile vs. remaining quartiles of self-reported distress about the child’s health, were performed using analysis of variance, adjusted for parental age, education, race, number of children in the family, extent of the child’s siblings’ illnesses, the child’s age, number of the child’s health problems, and type of surgery. Mean differences between child- and parent-reported CHRIS 2.0 measures by age of the child were compared using separate analyses of variance for each scale and composite measure.

### Missing data

The overall rate of missing data was < 10%, with < 5% of CHRIS 2.0 items missing. Given the limited amount of missing data, we carried out study analyses using only cases with complete data.

### IRB approval

The study was approved as minimal risk by the Institutional Review Board at the study site.

## Results

Characteristics of the 364 parent–child dyads included in this study appear in Table 1. The parents were moderately well-educated, approximately two-thirds were Hispanic, the majority were female (86.6%) and in their mid-30’s. Children were on average 7.5 years of age, with somewhat fewer females than males. Roughly 14% of the children in the study were under age 5. Including the participating child, there were approximately three children per family, with few 0.7 of the twelve chronic illnesses measured among siblings and roughly two of these twelve illnesses per participating child.

**Table 1:** Characteristics of the child and parent cohort samples^a^

	Child sample (*n* = 364)	Parents sample (*n* = 364)
Demographic characteristics		
Age (mean yrs)	7.5 [2.6]	36.7 [7.1]
Female (%)	40.7	86.6
Education (mean yrs)	2.3 [2.7]	13.3 [3.4]
Race/ethnicity (%)^b^		
Non-Hispanic white	24.1	28.2
Hispanic	68.1	63.3
Asian	6.0	6.2
African American	0.9	0.3
Children’s health problems		
Number of health problems^b^ (mean)		2.0 [2.7]
Family characteristics		
Number of children in family^b^	–	3.1 [1.6]
Other children’s illness^c^		0.7 [2.0]
Surgical type (%)		
ENT^d^	51.3	
Urology	16.4	
General	11.9	
Plastic	5.9	
Other	14.5	

^a^Table entries are means with standard deviations in parentheses or percentages as indicated; report by children and parents of 364 dyads from the pre-operative sample (and 146 dyads from the post-operative sample)

^b^Self-reported by parents

^c^Total number of chronic illnesses among participating child’s siblings (e.g., diabetes, asthma, weight problems, and allergies)

^d^Ear, nose, and throat

Internal consistency reliability coefficients for the CHRIS 2.0 subscales and composite measures for children and parents in this sample have been reported elsewhere [14]. Parents’ reports about their children’s health were consistently and significantly higher than children’s self-reports for each of the CHRIS 2.0 subscales and composite measures with the exception of overall quality of life (see Table 2). Children reported better CHRIS 2.0 overall quality of life scores than the parents’ reports about the child. The magnitude of these differences averaged approximately 60% of a standard deviation across CHRIS 2.0 measures. The level of agreement between parents and children, as indicated by the intraclass correlation coefficients, was < 0.40, a level considered as “poor” agreement [27] (see Table 2). Mental health measures had generally higher levels of parent–child agreement, compared with physical health measures. The magnitude of the differences between child- and parent-reported CHRIS 2.0 measures did not differ by age of the child (see Appendix A).

**Table 2:** Mean differences between *child*- and *parent*-reported CHRIS 2.0 measures (*n* = 364)^a^

CHRIS 2.0 measures	Reporter		Mean difference (± 95% CI)^b^		ES^c^	ICC^d^
	Child	Parent				
General health measures						
Physical function	61.9 [25.1]	80.1 [30.4]	− 18.2^***^	(− 21.8, − 14.6)	0.65	.09
Role function	67.1 [24.7]	86.7 [24.8]	− 19.6^***^	(− 23.0, − 16.3)	0.79	− .01
Social function	72.0 [25.9]	87.1 [23.9]	− 15.1^***^	(− 18.4, − 11.8)	0.61	.07
Cognitive function	67.3 [26.8]	87.6 [24.2]	− 20.3^***^	(− 23.8, − 16.8)	0.80	− .05
Energy	68.6 [22.7]	84.4 [27.1]	− 15.8^***^	(− 19.0, − 12.6)	0.63	.13
Pain^e^	74.2 [28.5]	80.5 [25.0]	− 6.2^***^	(− 9.3, − 3.2)	0.23	.36
Mental health	73.6 [25.3]	79.7 [14.0]	− 6.1^***^	(− 8.7, − 3.5)	0.31	.22
Overall quality of life	82.3 [20.6]	78.2 [20.2]	4.2^***^	(1.8, 6.5)	0.21	.36
Physical health composite	66.2 [20.6]	85.0 [23.3]	− 18.8^***^	(− 21.7, − 16.0)	0.86	.01
Mental health composite	74.0 [18.2]	79.9 [13.5]	− 6.0^***^	(− 7.7, − 4.2)	0.38	.39
Overall composite	69.3 [17.5]	82.0 [15.5]	− 12.7^***^	(− 14.6, − 10.8)	0.77	.20

*p < 0.05, **p < 0.01, ***p < 0.001

^a^Table entries are means with standard deviations in parentheses; scale scores have been transformed to range from 0 to 100 with high scores indicating better health

^b^Mean differences are entered as child-reported scores minus parent-reported scores for each measure with 95% confidence intervals for mean differences in parentheses; statistical significance is based on F-statistic using one-way ANOVA, adjusting for parental age, education, race, number of the child’s siblings, extent of sibling illness, child’s age, and number of the child’s health problems

^c^Effect size (mean difference expressed as a proportion of the pooled standard deviation)

^d^Intra-class correlation coefficients for agreement between child and parents scores on each measure (estimated using one-way random effects models and method of moments); scores < .40 are considered poor agreement^39^

^e^Pain was transformed such that higher score indicates less pain

Parents’ reports about their children’s health were significantly correlated with their reports about their own physical, mental, overall health, and quality of life (see columns 1–3, 5, Appendix B). We also observed significant correlations between parents’ reports about their children’s health and level of distress about their child’s health, *r* > 0.30, *p* < 0.01 (see column 4, Appendix B).

Compared to moderately and least distressed parents, the most distressed parents gave consistently lower ratings of their children’s health, across CHRIS 2.0 measures (see Table 3). They were thus closer to their children’s self-reports. These data have been adjusted for parental age, years of education, race/ethnicity, distress, quality of life, number of the child’s siblings, sibling illness, child’s age, and number of health problems. Of these variables in linear regression models assessing parent–child differences, only parental distress was significantly associated with differences in child–parent reports (data not shown).

**Table 3:** Mean differences between child- and parent-reported CHRIS2.0 measures for parents with higher/lower levels of distress (*n* = 352)^a^

CHRIS2.0 measures^b^	Least distressed^c^ (*n* = 113)	Moderately distressed^d^ (*n* = 159)	Most distressed^e^ (*n* = 80)	Adjusted *p*-value^f^
Physical function	− 27.4	− 18.6	− 9.1	0.009
Role function	− 26.4	− 18.4	− 13.5	0.009
Social function	− 21.9	− 12.7	− 10.9	0.011
Cognitive function	− 21.5	− 21.7	− 17.3	0.555
Energy	− 22.9	− 15.4	− 8.7	0.004
Pain	− 13.7	− 4.5	− 1.6	0.042
Mental health	− 4.6	− 5.8	− 9.8	0.793
Overall quality of life	− 0.2	3.3	9.7	0.194
Physical health composite	− 25.4	− 18.4	− 12.5	0.005
Mental health composite	− 8.6	− 5.5	− 4.3	0.105
Overall composite	− 17.5	− 12.3	− 8.3	0.002

^a^Measured using 4-item scale on the impact of the child’s physical and emotional health on parental well-being from the CHQ-PF28^30^

^b^Corresponding measures of each CHRIS2.0 construct reported by child and parent

^c^Reported as the lowest (least distressed) quartile of scores on the parental distress scale

^d^Reported as the intra-quartile of scores (moderately distressed) on the parental distress scale

^e^Reported as the highest (most distressed) quartile scores on the parental distress scale

^f^*P*-values based on ANOVA adjusting for parental age, education, race, number of siblings in the family, sibling illnesses, participating child’s age, and total number of participating child’s health problems

Parents scoring in the highest quartile of parental distress about their child’s health also gave substantially and statistically significantly lower ratings of their own mental health and quality of life compared to those scoring in the remaining quartiles (see Table 4). Compared to distressed parents, those with greater distress about their child’s health also reported poorer personal physical health, although this difference did not reach statistical significance. These analyses were also adjusted for parental age, education, race, number of children in the family, extent of sibling’s illness, the participating child’s age, and number of health problems.

**Table 4:** Personal health of most vs. less distressed parents (*n* = 352)^a^

Health measures	Parental distress^b^		Mean difference (± 95% CI)^e^
	Most distressed^c^ (*n* = 80)	Less distressed^d^ (*n* = 272)	
Physical health^f^	82.8 [3.5]	87.3 [2.7]	− 4.5^§^ (− 10.6, 1.5)
Mental health^g^	61.7 [2.4]	75.9 [1.9]	− 14.2^***^ (− 18.3, − 10.0)
Quality of life^h^	63.3 [3.2]	74.3 [2.5]	– 11.0^***^ (− 16.6, − 5.3)

§*p* = .141

***p < .001

^a^Table entries are adjusted means with standard errors in parentheses based on analysis of covariance adjusted for parental age, education, race, number of siblings in the family, sibling illnesses, child’s age, and number of health problems

^b^Parental distress was measured using 4 items from the CHQ-Parent Form 28^30^ related to impact of the child’s health on the parent’s emotional well-being and time for personal needs

^c^Parents scoring in the highest quartile of parental distress

^d^Parents scoring in the 3 quartiles of parental distress other than the highest, combined

^e^Mean difference calculated as less minus more distressed parent scores for each health measure, with 95% confidence intervals around mean differences

^f^Measured as a composite of the 7-item CHRIS2.0 parent physical function scale

^g^Measured as a composite of the 10-item CHRIS2.0 parent mental health scale

^h^Measured using the 8-item QLRS^35^

## Discussion

Consistent with a substantial body of existing literature [5, 6, 16, 28], we found significant differences between parents’ reports about their children’s health and children’s self-reports using the CHRIS 2.0 measure. The perspective parents in our study adopted, as a proxy reporter or from their own view of the child’s health, was not explicitly addressed. As noted by Snow AL et al. [12], the definition of a ‘proxy’ is data “collected from someone who speaks for a patient who cannot, will not, or is unavailable to speak for him or herself” and is therefore attempting to provide information on the same construct as would the patient if s/he were able to provide that information themselves. It is not clear whether the poor agreement between parent and child reports of the child’s health across multiple studies suggests that parents were acting as proxy or other rater reporters about the child’s health. Our instructions to parents were only to ask them to rate their child’s health with the implicit assumption that they were acting as proxy reporters. It would be expected therefore that there should be similarities between the two respondents if both perspectives are obtained. But as Pickard AS, et al., 2005 [29] has suggested, the instructions given to the proxy, including to “assess the patient as they think the patient would respond” vs. provide their own perspective on the patients health (i.e., same construct, different perspective), would perhaps provide a valuable clarifying context for proxy reporting in future research.

Also consistent with other research we found that parents’ assessments of their children’s health were positively correlated with reports of their own health [5, 30–32]. We found that parents who were in greater distress about their child’s health also reported poorer personal health, but were closer to their child’s self-reports.

That parents and children differ in assessments of the child’s health is supported by a number of studies and systematic reviews that span a broad spectrum of diseases, populations, and clinical settings [1–7]. Most of these studies used well-tested measures of young children’s health-related quality of life, such as the Pediatric Quality of Life Inventory (PedsQL) [28], the Functional Disability Inventory (FDI) [33], KIDSCREEN [34], and the Child Health Questionnaire (CHQ) [35], that require interviewer assistance or parent report for children who cannot read. Unique to this study is the use of the CHRIS measure for children as young as 4 years of age to self-report their health-related quality of life. Such self-reporting, independent of parents or interviewers, can avoid the potential proxy-reporting bias observed for children and adults [13, 16, 29, 36].

Parents in our study over-estimated their child’s self-reported health. This finding is consistent with other studies of parent–child differences among “healthy” children [1, 5]. Although children in our sample were about to undergo surgery, the majority did not have chronic diseases and surgeries or procedures were outpatient or not serious, making them more similar to a “healthy” child sample.

We also observed fewer parent–child differences for mental vs. physical health measures. This finding was not consistent with the hypothesis that parents were better at approximating the child’s self-reports for more “observable” behaviors [4, 5, 9]. It may be that the animation of content afforded by the CHRIS measure more effectively communicated the feelings and emotions related to the mental health items or that parents were more attuned to the emotional state of their children in the perioperative period. One feasibility study in the UK of animation vs. text presented by tablet or on paper assessing children’s self-reported health utilities, administered to children ages 4–14 in school and hospital settings, found that children preferred animation over either text presentation and even the youngest age group were able to understand the animated questions [37]. More research is needed to explore the impact of animation on parent–child differences in health-related quality of life. We did not find evidence of fewer or greater differences between children and parents by the age of the child.

The finding that parents report about their child were positively correlated with their personal health supported the “self-referencing” hypothesis proposed by some researchers [5, 36]. That is, in reporting about the health of another, proxies use their own health as the referent. In comparisons among adults, for example, proxies appear to report lower health-related quality of life scores (or under-estimate health) for elderly patients than the elderly themselves [36]. In contrast, as noted above, parents of healthy children tended to over-estimate the child’s health, especially for young children [6].

Although cited as a need for further research [3, 5, 6], few studies have empirically examined the factors contributing to parent–child differences in reporting health-related quality of life. We found that, adjusted for demographic characteristics of children and parents and the number and severity of health problems of the participating children and their siblings, parental distress was the principal contributor to parent–child differences in CHRIS 2.0 scores. Further, compared to less distressed parents, those reporting greater distress over their child’s health were closer to the child’s self-reported CHRIS 2.0 scores. This finding is consistent with other studies among parents of children with chronic diseases [11, 30, 38, 39].

A few studies of parent–child differences in reports of children’s health have found similar results, namely more distressed parents report lower scores for their child’s health [10, 32, 40]. Our study was not designed to explore the causal direction of this relationship. The illness of a child could certainly cause some parents greater distress than others or the attentiveness required to attain a better understanding of the child’s perception of their health could cause greater parental worry or concern and limit their attention to their own needs. Distressed parents in our study also reported poorer personal health, raising questions and concerns about the toll “vigilant parenting” may be taking on parents [38]. This toll could be greater for parents of children with chronic diseases [10, 38]. Understanding the extent of the gap between parent and child perspectives on the child’s health, the relationship of parental distress to that gap and the effects of parental distress on the overall health of parents as caregivers could have important implications for effective and family-centered pediatric care [40].

As the pace and scope of interventions to improve pediatric care has accelerated, the need to evaluate their effectiveness, not using only clinical and biomarkers of health, but also their impact on children’s health-related quality of life has been increasingly recognized [2]. The CHRIS 2.0 measure is a reliable and valid health-related quality of life measure [14, 15] and allows young children to report their health independent of adults in busy clinical settings.

Despite this independent reporting, our study highlighted not only the persistence of parent–child differences in perceptions of children’s health, but also the importance of parental distress related to both the magnitude and direction of these differences. If, as recommended by some, differences between parent and child are not as biased or inaccurate in reporting by one or the other, but rather as a “quantifier of the gap” [12], including both could provide a more developed or comprehensive picture of the child’s health. Exploring the nature of that gap during office visits, including parallel assessments of parental distress over the child’s health, could have important implications for both effective clinical care for the child [2–4, 16], for family–physician communication, for family-centered pediatric care [41, 42], and for parents’ health and well-being. Integration of measures of children’s health-related quality of life, such as the CHRIS 2.0, and parent’s health as additional “vital signs” in routine pediatric practice [43] could provide valuable and unique information for clinicians and researchers evaluating and tailoring treatment regimens and improving pediatric practice.

### Limitations

This study was conducted at a single pediatric academic healthcare institution. Whether the findings of this study replicate in other healthcare settings requires additional study. Also, although the study sample was highly diverse, some racial/ethnic groups, including African Americans, were underrepresented. Differences in parent–child reports of children’s health in other such racial and ethnic groups will require additional study. We also studied children about to undergo elective surgical procedures. Although our prior work suggests the current findings replicate among children with chronic conditions [15], CHRIS 2.0 will require additional testing in populations of children with other conditions and among “healthy” children in the included age range. Finally, in a recent study [14], we tested the CHRIS 2.0 among children as young as 4 years of age and found the reliability of their reports to parallel those for older age groups. In this study we also observed that differences in parent–child reports for the youngest children also paralleled the findings for older age groups. We do not know whether reliability findings and parent–child differences for even younger children would produce findings observed for this study. Finally, we did not directly assess whether and to what extent parents assisted children to complete the CHRIS2.0 measure at home. However, to the extent that parents influenced the child’s reports, more alignment of responses would have been expected, which we did not find.

## Conclusion

The differences between parent and child in reports about the child’s health are striking and suggest an opportunity for clinicians to explore the nature of those differences. The CHRIS 2.0 offers an opportunity to include the child’s voice in the direct reporting of their own health-related quality of life without assistance from adults. It can produce reliable and valid results even among young children. In light of the finding that more distressed parents provided assessments that were closer to the child’s self-reports, exploring the nature and potential cost of parental vigilance on the parents’ own health could also provide valuable insight for the clinicians caring for these children and their families. Further studies are needed to assess the generalizability of CHRIS 2.0 in other health settings and conditions and to other racial/ethnic groups.

## Supplementary Information

Below is the link to the electronic supplementary material.

Supplementary file1 (DOCX 16 kb)

Supplementary file2 (DOCX 23 kb)
