# Supplementary material for: The role of parental health and distress in assessing children’s health status
Source: Qual Life Res. 2022 Jul 25;31(12):3403–12. doi: 10.1007/s11136-022-03186-z (PMC9587925; doi:10.1007/s11136-022-03186-z)
Supplement: Supplementary file 1 — Supplementary file1 (DOCX 16 kb) [file 11136_2022_3186_MOESM1_ESM.docx]

| **Appendix A. Mean differences between *child*-reported and *parent*-reported CHRIS2.0 measures by child's age (n=338)^1^** | | | | | | | | | | |
| --- | --- | --- | --- | --- | --- | --- | --- | --- | --- | --- |
| CHRIS2.0 measures^2^ | Child's age | | | | | | | | | |
|  | 4 (n=46) | 5 (n=48) | 6 (n=44) | 7 (n=37) | 8 (n=35) | 9 (n=34) | 10 (n=35) | 11 (n=28) | 12 (n=31) | p-value^3^ |
| ·   Physical function | -17.1 [38.5] | -21.1 [40.2] | -30.7 [34.9] | -19.8 [35.5] | -19.3 [29.4] | -17.6 [29.0] | -11.1 [36.6] | -6.0 [36.3] | -11.7 [25.5] | 0.151 |
| ·   Role function | -18.8 [36.8] | -20.1 [36.7] | -18.8 [37.7] | -26.7 [34.7] | -19.5 [23.2] | -19.9 [23.4] | -20.0 [34.3] | -21.9 [28.4] | -12.5 [23.4] | 0.899 |
| ·   Social function | -13.3 [34.8] | -16.9 [38.5] | -19.3 [36.1] | -17.9 [29.1] | -17.1 [22.5] | -15.4 [28.2] | -12.5 [35.1] | -9.8 [25.3] | -7.7 [31.7] | 0.851 |
| ·   Cognitive function | -17.4 [36.7] | -20.1 [36.4] | -25.9 [48.8] | -23.6 [27.3] | -23.9 [27.8] | -22.1 [30.6] | -14.6 [38.3] | -16.1 [18.9] | -14.9 [26.5] | 0.817 |
| ·   Energy | -7.1 [28.9] | -22.4 [39.5] | -11.0 [32.4] | -19.1 [24.0] | -19.8 [20.3] | -22.1 [34.6] | -11.0 [35.1] | -14.6 [24.9] | -20.2 [25.3] | 0.205 |
| ·   Pain | -1.6 [22.8] | -15.1 [38.5] | -13.1 [32.6] | -4.7 [23.5] | -5.0 [20.8] | -6.6 [34.4] | -5.7 [23.6] | -2.7 [29.1] | 4.8 [31.9] | 0.126 |
| ·   Mental Health | 0.6 [22.3] | -5.0 [27.3] | -8.6 [34.3] | -1.6 [21.3] | -8.6 [28.6] | -8.9 [24.1] | -14.8 [22.1] | -15.8 [21.0] | 0.3 [18.2] | 0.048* |
| ·   Overall quality of life | 10.6 [22.0] | 1.3 [24.4] | 7.1 [22.8] | 3.4 [17.3] | 5.4 [19.5] | -0.4 [26.4] | 5.0 [26.8] | 3.1 [20.9] | 0.8 [24.6] | 0.505 |
| *·   Physical health composite* | -17.4 [32.5] | -20.2 [32.7] | -24.1 [30.8] | -22.8 [27.5] | -20.1 [18.9] | -19.0 [22.1] | -15.4 [33.1] | -14.2 [22.8] | -12.0 [21.2] | 0.668 |
| *·   Mental health composite* | 1.7 [11.9] | -10.6 [21.7] | -6.7 [21.0] | -4.1 [12.3] | -5.2 [13.1] | -10.2 [17.9] | -7.2 [18.6] | -9.5 [15.3] | -4.7 [14.9] | 0.032* |
| *·   Overall composite* | -8.9 [20.0] | -16.3 [20.9] | -16.2 [20.6] | -14.0 [17.5] | -12.1 [12.8] | -14.3 [15.6] | -11.8 [21.0] | -11.6 [17.2] | -7.5 [12.9] | 0.369 |
| ^1^Table entries are means with standard deviations parentheses, reported as mean differences (child minus parent reports) for CHRIS2.0 scores for each subscale and 3 composites by age group ^2^Reported separately for child and parent for each child-parent dyad (n=338) ^3^Based on F-value form separate ANOVA for each subscale and composite *Non-significant after Bonferroni correction | | | | | | | | | | |
