# Supplementary material for: The role of parental health and distress in assessing children’s health status
Source: Qual Life Res. 2022 Jul 25;31(12):3403–12. doi: 10.1007/s11136-022-03186-z (PMC9587925; doi:10.1007/s11136-022-03186-z)
Supplement: Supplementary file 2 — Supplementary file2 (DOCX 23 kb) [file 11136_2022_3186_MOESM2_ESM.docx]

**Appendix B. Correlation of *parent*-reported baseline CHRIS2.0 measures of child’s health with parent’s own health (n=364)^1^**

| *Parent*-reported  CHRIS2.0 measures of child’s health | *Parent*-reported personal health-status measures | | | | |
| --- | --- | --- | --- | --- | --- |
|  | (1) Physical health composite^2^ | (2) Mental health composite^3^ | (3) Overall composite^4^ | (4) Parental distress^5^ | (5) QLRS-P^6^ |
| - CHRIS 2.0 Physical health composite | .30^***^ | .14^**^ | .27^***^ | -.31^**^ | .00 |
| - CHRIS 2.0 Mental health composite | .26^***^ | .40^***^ | .39^***^ | -.50^***^ | .35^***^ |
| - CHRIS 2.0 Overall composite | .32^***^ | .29^***^ | .37^***^ | -.45^***^ | .18^***^ |
| - QLRS-C^7^ | .22^***^ | .35^***^ | .33^***^ | -.35^***^ | .51^***^ |

^*^p<0.05
 ^**^p<0.01

^***^p<0.001

^1^Table entries are Pearson product correlation coefficients

^2^7-item composite of physical function, role function, social function, cognitive function and energy/vitality, paralleling the CHRIS2.0 child physical health composite; scores range from 0-100

^3^10-item composite of 5 items from the CES-D 20-item measure^31,32^ and the 5 item mental health measure from the SF-36^33,34^, paralleling the content for the CHRIS2.0 mental health composite scores, ranging from 0-100, with higher scores meaning better health

^4^The overall composite for the parent-reported personal health status measure combines physical and mental health composites into a single score, ranging from 0-100, with higher scores meaning better health

^5^A composite 4-item measure of parental distress based on frequency of worry over or burden from a child’s physical or emotional health, rated on a 5-point Likert scale, ranging from “All of the time” to “None of the time,” transformed to range from 0-100 with high scores meaning more distress^30^

^6^The quality of life rating scale for parents (QLRS-P) is an 8-item measure asking parents to rate their quality of work life, family life, friendships, sex life, daily routine, health personal/leisure/social item and general life on a 5-point Likert scale ranging from “Excellent” to “Poor,” with higher scores meaning better quality of life^35^

^7^The quality of life rating scale for children (QLRS-C) is a composite 8-item measure of the *parent’s* ratings of the child’s physical health, mental health, family life, friendships, school work, self-confidence, playtime or fun, and general life enjoyment on a 5-point Likert scale ranging from “Excellent” to “Poor,” transformed to range from 0-100 with higher scores meaning higher quality of life
